# Vinorelbine-based salvage therapy in HER2-positive metastatic breast cancer patients progressing during trastuzumab-containing regimens: a retrospective study

**DOI:** 10.1186/1471-2407-8-209

**Published:** 2008-07-24

**Authors:** Filippo Montemurro, Stefania Redana, Franco Nolè, Michela Donadio, Maria Elena Jacomuzzzi, Giorgio Valabrega, Giuseppe Viale, Anna Sapino, Massimo Aglietta

**Affiliations:** 1 Divisione di Oncologia Medica, AO Ordine Mauriziano/Istituto per la Ricerca e la Cura del Cancro, Candiolo, Torino, Italy; 2 Dipartimento di Oncologia Medica, Istituto Europeo di Oncologia (IEO), Milano, Italy; 3 Centro Oncologico Subalpino (COES), Torino, Italy; 4 Divisione di Ginecologia Oncologica, AO Ordine Mauriziano/Ospedale Mauriziano Umberto I, Torino, Italy; 5 Dipartimento di Patologia e Medicina di Laboratorio, Istituto Europeo di Oncologia (IEO), Milano, Italy; 6 Dipartimento di Scienze Biomediche e Oncologia Umana, Ospedale San Giovanni Battista- Molinette, Torino, Italy

## Abstract

**Background:**

The vinka-alkaloyd vinorelbine is a potentially valuable treatment in patients with HER2-positive, trastuzumab-resistant advanced breast cancer. We sought to document the clinical activity of vinorelbine-based salvage treatments in this clinical setting.

**Methods:**

We analyzed a cohort of 424 consecutive women receiving trastuzumab-based therapy for HER2-positive advanced breast cancer. Of these, 299 were identified as progressing during the initial trastuzumab-based treatment, and 77 received vinorelbine-based therapy as first salvage treatment. Central review of pathological specimens revealed that 70 patients had HER2-amplification detected by FISH. For these patients we determined overall response rate (ORR = complete-CR + partial-PR) and clinical benefit (CB = CR+PR+ Stable disease lasting at least 6 months), time to progression (TTP) and overall survival (OS) from the initiation of vinorelbine-based salvage therapy.

**Results:**

In 60 patients who were evaluable for tumor response, ORR and CB rates were 28% (95% C.I. 18%-41%) and 50% (95% C.I. 38%-62%), respectively. Median follow-up from the initiation of salvage therapy was 15 months (range 1–63 months). Median TTP and OS were 7.1 months (95% C.I. 6.6–7.7 months) and 21 months (95% C.I. 14.3–27.7 months), respectively. No differences in clinical outcomes were observed according to whether vinorelbine was administered as a single agent or in combination with other cytostatics, or whether trastuzumab was stopped or continued beyond disease progression.

**Conclusion:**

our findings suggests that vinorelbine-based combinations are active and should be further evaluated in studies conducted in trastuzumab-resistant patients, including those evaluating newer HER2-targeting agents.

## Background

Trastuzumab is a monoclonal antibody directed against the product of the *HER2/neu *oncogene, which is amplified in about 20–25% of breast cancer cases.[1] The combination of trastuzumab and chemotherapy resulted in improved clinical outcomes, compared with chemotherapy, alone, in patients with HER2-positive advanced breast cancer.[2,3] Because of unacceptable rates of cardiac toxicity when trastuzumab was given in combination with anthracycline-based therapy,[2] this monoclonal antibody was registered for the treatment of HER2-positive advanced breast cancer patients with the taxanes paclitaxel and docetaxel. Furthermore, several phase II trials have been conducted using alternative regimens, which were based on preclinical observations suggesting additivity or even synergism between trastuzumab and other commonly used cytostatic agents. [4,5] One of these compounds is vinorelbine, a vinka-alkaloyd derivative that has shown remarkable clinical activity in anthracycline-pre-treated advanced breast cancer patients.[6,7] This drug is available both as intravenous and oral formulations.[8] Preclinical synergism between vinorelbine and trastuzumab was partially confirmed in the clinic in the context of phase II trials, where up to 84% response rates were reported when vinorelbine and trastuzumab were used as first-line treatment in appropriately selected advanced breast cancer patients. [9-12] Due to its intrinsic antitumor activity and positive interaction with trastuzumab, vinorelbine is a suitable salvage choice in HER2-positive advanced breast cancer patients whose disease has progressed during an initial trastuzumab-based regimen. Before the availability of the dual epidermal growth factor receptor (EGFR) and HER2 tyrosine kinase inhibitor lapatinib,[13,14] treatment options in trastuzumab-resistant patients included salvage chemotherapy with or without the continuation of trastuzumab. [15-18] A recently published randomized phase III trial with lapatinib and capecitabine confirms that HER2 is still an exploitable target after trastuzumab-failure, opening the way to newer treatment options in this setting.[13]

With the current retrospective analysis, we sought to describe patterns of use and clinical activity of vinorelbine-based salvage therapy in trastuzumab-resistant patients using data collected before the availability of lapatinib. In the absence of data from prospective trials, our aim is to evaluate the potential worth of vinorelbine as a component of salvage strategies including newer HER2-targeting agents in trastuzumab-resistant patients.

## Methods

### Patients

Patients for this analysis were selected from a database including 424 consecutive women with HER2 positive advanced breast cancer who received trastuzumab-based therapy for the treatment of advanced disease between September 1999 and April 2007 at 11 different Institutions in Italy, United Kingdom and Hungary (see acknowledgments). No patients had previously received trastuzumab as part of adjuvant treatment for operable disease. For patients developing tumor progression during the initial trastuzumab-based regimen, investigators at each site were asked to provide details of the first post-progression treatment (drugs and doses, best tumor response, date of further progression, and date of death or of last follow-up visit). This study was not prospectively planned. Thus, salvage treatments were indicated by treating physicians at each Institution. Follow-up information was updated as of December 2007.

Being a retrospective analysis based on patients treated according to standards of care, no specific informed consent was obtained. However, data collection and provision was conducted in compliance with the ethical requirements of each of the participating Institutions.

### Statistical methods

Tumor response was recorded by treating physicians at each site according to the World Health Organization criteria.[19] Response rate was defined as the proportion of patients achieving complete or partial remission (CR+PR). Imaging studies were not available to centrally reassess response rates. Patients with disease that was confined to the bone or who had effusions as the only evidence of metastatic disease were considered non evaluable for response, as were patients with isolated central-nervous disease progression. For all patients time-to progression (TTP) and overall survival (OS) were calculated by the Kaplan Meyer method starting from the date of salvage therapy start.

Comparisons between categorical variables evaluated by the Chi Square or the Fisher's exact test. Survival curves were compared by the log-rank test. Statistical significance was set at p < 0.05. All the analyses were conducted by the SPSS 15.0 statistical package

## Results

We identified a total of 299 patients progressing during the initial trastuzumab-based treatment. Of these patients, 77 received vinorelbine-based therapy as first salvage treatment. We excluded 7 patients because their HER2 status was either 2+ without further evaluation of HER2 amplification, or because HER2 positivity had been defined by unconventional methods (i.e. percentage of positive cells) and tissue blocks were unavailable for reassessment. For the remaining 70 patients, central assessment by fluorescence in situ hybridization (FISH) confirmed *HER2/neu *amplification in their tumor material.

Patients selected for the analysis had previously received trastuzumab combined with weekly (2) or three-weekly (31) docetaxel, weekly (12) or three-weekly (5) paclitaxel, three-weekly docetaxel plus epirubicin (4), weekly docetaxel plus carboplatin (2), capecitabine (1), infusional 5-fluorouracil (1), vinorelbine plus infusional 5-fluorouracil (1), and vinorelbine alone (4). These latter 5 patients had stopped vinorelbine-based therapy before disease progression either because of complete tumor response (1 patient), long-lasting stable disease which was maintained with trastuzumab alone (3 patients), or toxicity determining treatment discontinuation (1 patient). Additionally, 4 patients had received trastuzumab alone, whereas for 3 patients the initial trastuzumab-containing regimen was not known.

Response rate (CR+PR) to the initial trastuzumab-containing regimen was 55% in 69 evaluable patients. Median tumor progression during the initial trastuzumab-containing regimen was 8.6 months (95% C.I. 7.5 to 9.6 months).

Patient characteristics before the initiation of vinorelbine-based salvage treatment are summarized in Table 1. Notably, most of the patients had visceral involvement and a substantial proportion had metastatic central nervous system involvement. Vinorelbine-based salvage treatments are summarized in Table 2. Trastuzumab was continued beyond disease progression in 36 patients (51%) and stopped in the remaining 34 patients (49%). In 60 evaluable patients, the overall response rate was 28% (95% C.I. 18%-41%) (Table 3). Additionally, 13 patients experienced disease stabilization that lasted 6 months or longer, for a clinical benefit rate of 50% (95% C.I. 38%-62%). Response and clinical benefit rates did not differ significantly according to whether patients received single agent vinorelbine or vinorelbine combined with other agents, or whether trastuzumab was continued or not beyond disease progression. Of the 5 patients who were re-treated with vinorelbine-based therapy, 1 achieved a partial response, two achieved a stable disease lasting 6.1 and 3.2 months, and two had disease progression. Of the 39 evaluable patients who underwent monochemotherapy with vinorelbine, 13 received chemotherapy alone and 26 continued trastuzumab beyond progression with vinorelbine. ORR was 38% (95% C.I 18%-64%) and 15% (95% C.I. 6%-34%) according to whether trastuzumab was stopped or continued, respectively, but this difference was not statistically significant (two-tailed Fisher's exact test, p = 0.13). Due to the small number of patients involved, these groups were not submitted to further analysis of clinical outcomes.

**Table 1:** Patient characteristics (n 70)^1^

Variable	Values (%)^2^
Median Age in years (range)	55 (32–77)
Stage at Initial Diagnosis of Breast Cancer	
I/II	41 (59)
III	17 (24)
IV	12 (17)
Hormone Receptor Status	
ER and or PgR positive	33 (47)
ER and PgR Negative	35 (50)
ER and PgR Unknown	2 (3)
Exposure to anthracycline before the initial T-based regimen^3^	46 (66)
Exposure to taxane before the initial T-based regimen^3^	18 (26)
Prior Lines of Chemotherapy for Metastatic Disease^4^	
1	48 (68)
2	10 (14)
≥ 3	12 (17)
Initial Trastuzumab-based regimen	
Docetaxel 75–100 mg/m^2 ^q3wks + T	31 (44)
Paclitaxel 80–90 mg/m^2 ^weekly +T	12 (17)
Paclitaxel 175 mg/m^2 ^q3wks + T	5 (7)
Vinorelbine 25–30 mg/m^2 ^weekly + T	4 (6)
Docetaxel 75 mg/m^2 ^+ Epi-doxorubicin 75 mg/m^2 ^q3wks + T	4 (6)
T alone	4 (6)
Other regimens	7 (10)
Unknown	3 (4)
Median Number of Metastatic Sites	2 (1–6)
Pattern of Metastatic Disease	
Visceral (Liver + Lung)	57 (81)
Non Visceral/Non CNS (Bone, Soft Tissue, Effusions)	11 (16)
Central Nervous System (plus other sites)	17 (24)

T, Trastuzumab; CNS, central nervous system.

^1^All patients had HER2 amplification confirmed by central reassessment of tumor material by fluorescence *in situ *hybridization

^2^Because of rounding, the sum of percentages does not always equal 100.

^3^Exposure to these drugs in the neoadjuvant, adjuvant or metastatic setting before the initial trastuzumab-based regimen.

^4^Including the initial trastuzumab-based regimen

**Table 2:** Vinorelbine-based salvage treatments

Type	Number	%*
Vinorelbine alone	16	23
Vinorelbine + T	32	46
Vinorelbine + Infusional 5-FU or Capecitabine	8	11
Vinorelbine + Infusional 5-FU or Capecitabine + T	3	4
Vinorelbine + Gemcitabine	8	11
Vinorelbine + other agents**	3	4

Legend: T, trastuzumab

*Because of rounding sum of percentages does not equal 0.

**1 patients in this group received also trastuzumab

**Table 3:** Summary of response rates to vinorelbine-based salvage treatments

Response	Overall	Single agent vinorelbine	Vinorelbine + other chemotherapies	Continuing trastuzumab	Stopping Trastuzumab
	N60	N 39^1^	N 21	N 29	N 31
ORR	17 (28%)	9 (23%)	8 (38%)^2^	6 (21%)	11 (36%)^3^
CR	1 (2%)	0 (0%)	1 (5%)	0 (0%)	1 (3%)
PR	16 (27%)	9 (23%)	7 (33%)	6 (21%)	10 (32%)
SD	16 (27%)	12 (31%)	4 (19%)	8 (28%)	8 (26%)
PD	27 (45%)	18 (46%)	9 (43%)	15 (52%)	12 (39%)
					
CB (CR+PR+SD ≥ 6 months)	30 (50%)	15 (38%)	11 (52%)	13 (48%)	18 (58%)

ORR; overall response rate (CR+ PR); CR, complete remission: PR, partial response; SD, stable disease; PD, progressive disease; CB, clinical benefit

^1^26 of these patients received single-agent chemotherapy with vinorelbine and continued trastuzumab beyond progression

^2^Two tailed Fisher's Exact test for the difference in ORR (CR+PR) = 0.24

^3 ^Two tailed Fisher's Exact test for the difference in ORR (CR+PR) = 0.26

**Figure 1:**
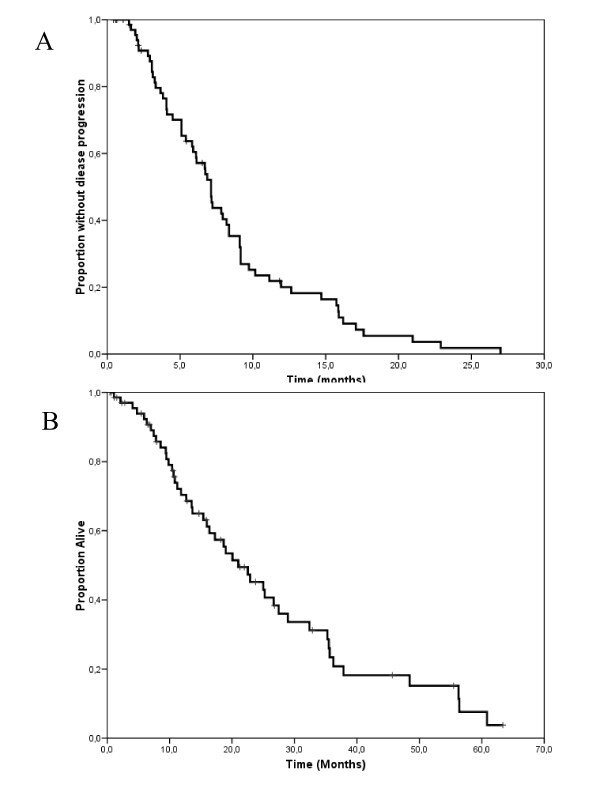
Kaplan-Meyer estimates of time-to progression (a) and overall survival (b) for 70 patients selected for the analysis.

**Figure 2:**
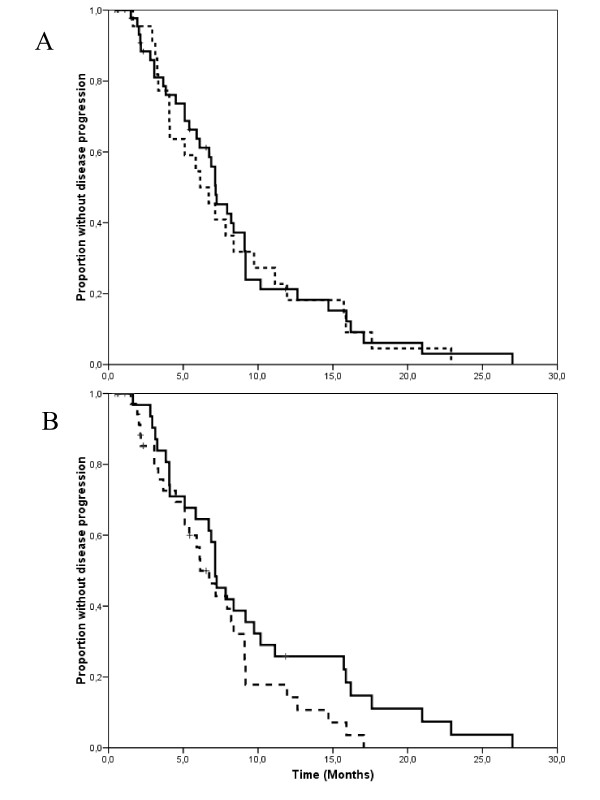
**a. Kaplan-Meyer estimates of time-to progression according whether vinorelbine was given alone (solid line) or combined with other agents (dashed line).** Median time-to progression was 7.2 and 6.1 months for patients receiving single agent vinorelbine or vinorelbine with other chemotherapy agents (log-rank test, p = 0.76). b. Kaplan-Meyer estimates of time-to progression according whether trastuzumab was stopped (solid line) or continued (dashed line) during salvage treatment with vinorelbine. Median time-to progression was 7.1 and 6.1 months for patients stopping or continuing trastuzumab, respectively (log-rank test, p = 0.10).

**Figure 3:**
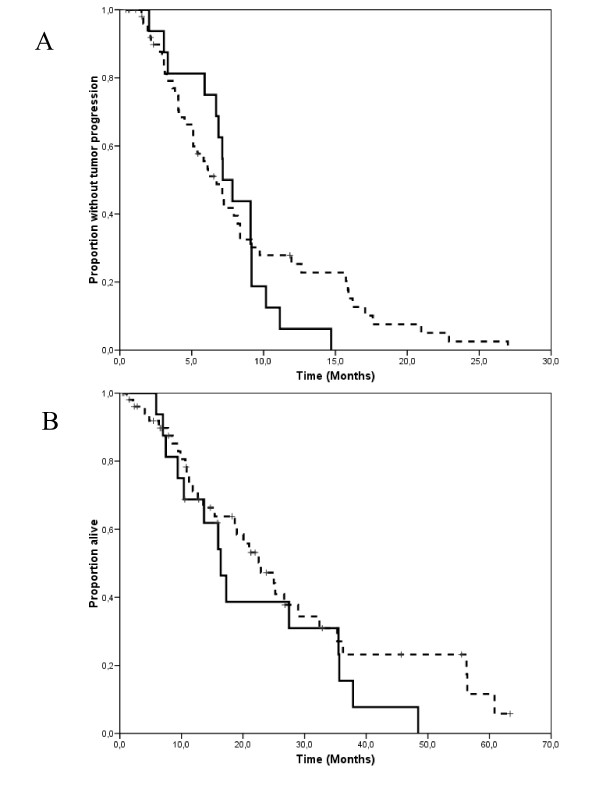
**Kaplan-Meyer estimates of time-to progression (a) and overall survival (b) for patients with (solid line) or without (dashed line) central nervous system metastases.** Median time to progression was 7.1 and 6.7 months for patients with and without central nervous system involvement, respectively (long-rank test, p = 0.51). Median overall survival was 16.4 and 22.9 months for patients with and without central nervous system involvement, respectively (log-rank test, p = 0.24)

At a median follow-up measured from the initiation of salvage treatment of 15 months (range 1–63 months), 60 patients had progressed and 46 had died because of tumor progression. Median TTP and OS measured from the initiation of salvage treatment were 7.1 months (95% C.I. 6.6–7.7 months) and 21 months (95% C.I. 14.3–27.7 months), respectively (Figure 1a and 1b). Time-to progression did not differ substantially according to whether patients received single-agent vinorelbine, or vinorelbine combined with other cytostatics (Figure 2a), or whether trastuzumab was continued or stopped (Figure 2b). The 17 patients with central nervous system involvement were managed with radiation therapy (either whole brain, stereotactic irradiation or both) as part of multimodal treatment. All these patients except one had had central nervous system progression during the initial trastuzumab-based therapy. Interestingly, their time-to progression and overall survival measured from the initiation of salvage with vinroelbine-based therapy did not differ substantially from that of patients without central nervous system involvement (Figure 3a and 3b).

## Discussion

Our retrospective analysis shows that vinorelbine-based therapy is a valuable salvage option for patients with advanced HER2-posive (*HER2/neu *amplification) whose disease has progressed during an initial trastuzumab-based regimen. A clinical benefit rate of 50% and a median time-to progression of about 7 months are noteworthy, considering that a high proportion of these patients had visceral involvement and about a quarter had central nervous system metastases. Having been generated from a retrospective analysis, our results should be evaluated with caution because of the well known biases concerned with this methodology. For example, tumor response was recorded by each investigator at each site and no centralized review was performed. Furthermore, in the estimation of time-to progression, it must be considered that timing of disease assessment was not pre-planned and uniform between institutions. However, we believe that our findings represent a reasonable estimation of the clinical activity of vinorelbine-based salvage regimens in the daily clinical practice. Cross-comparisons of data generated from retrospective analyses with that of prospective studies are not appropriate in general, but may serve to evaluate the plausibility of our findings. Currently, only two prospective, randomized trials have provided data on the treatment of patients with trastuzumab-resistant disease. One is the already mentioned study with the dual EGFR/HER2 inhibitor lapatinib.[13] This study, conducted in anthracycline and taxane pre-treated HER2-positive advance breast cancer patients whose disease was clinically resistant to trastuzumab, was closed after the first interim analysis reporting an about 50% reduction in the risk of relapse for patients receiving lapatinib plus capecitabine, compared with capecitabine alone. This translated in an about 4 months improvement in median time-to progression (from 4.4 to 8.4 months).

Response rate was 14% with capecitabine alone and 22% with the addition of lapatinib to capecitabine. As a result of this trial, lapatinib plus capecitabine is now an accepted option for trastuzumab resistant patients.[20]

Another study sought to evaluate the worth of continuing trastuzumab beyond disease progression in combination with capecitabine, compared with capecitabine alone in patients who had failed first-line trastuzumab-based therapy.[21] This study was a valuable attempt at settling the ongoing controversy of the worth of continuing trastuzumab when disease progresses during an initial trastuzumab-based treatment. Unfortunately, due to slow accrual, the study was closed prematurely when it had randomized only 156 of the 482 planned patients. In this small cohort, response rate was 24.6% and 49.1% in patients who stopped and continued trastuzumab, respectively. Continuation of trastuzumab was also associated with a 30% reduction in the risk of tumor progression, translating in an about 3-month increase in median time to progression, from 5.6 to 8.5 months. Our findings provide a suggestion that vinorelbine-based salvage therapy is associated with a median time to progression that might be close to that of the experimental arms of these two randomized trial. On account of these results, vinorelbine-based therapy should be evaluated in studies conducted in trastuzumab-resistant patients, including those evaluating newer HER2-targeting agents.

Another limitation to the applicability of these results is the fact that a substantial number of HER2-positive advanced breast cancer patient receive vinorelbine as part of the first-line trastuzumab-based therapy in the clinical practice. In a previous comprehensive analysis of our database, we observed that this option was pursued in about 38% of patients, compared with 56% receiving a taxane + trastuzumab (Montemurro et al, in press). Although data leading to the registration of trastuzumab were originated from prospective randomized studies with taxanes,[2,3] vinorelbine and trastuzumab-based regimens have become popular because of high efficacy documented in phase II trials and a favorable toxicity profile. This, apart from treating physicians' preferences towards salvage regimens, partly explain the reason why only 26% of patients that we identified as progressing during an initial trastuzumab-based regimen received vinorelbine-based salvage therapy. It must be noted, however, that due to the increasing use of taxanes and trastuzumab in the adjuvant setting, vinorelbine qualifies as a valuable option in recurring patients, whose disease fulfils the criteria of trastuzumab resistance.

Finally we could not find significant differences in clinical outcomes between vinorelbine-based strategies (i.e. continuing or stopping trastuzumab beyond disease progression or adding another agent to vinorelbine).

## Conclusion

Our analysis suggests that vinorelbine-based therapy has encouraging clinical activity in patients with trastuzumab-resistant HER2-positive breast cancer and might represent a platform for future developments in this clinical setting.

## Competing interests

The authors declare that they have no competing interests.

## Authors' contributions

FM conceived the study, performed statistical analyses, and wrote the manuscript. SR participated in designing the study, collected clinical data and collaborated in the writing of the manuscript. FN provided clinical data and revised the manuscript. MD provided clinical data and revised the manuscript. MEJ provided clinical data. participated in designing the study, collected clinical data. GVa participated in designing the study and collected clinical data. GVi provided clinical data and revised the manuscript. AS performed centralized analyses of HER2 amplification status. MA revised the manuscript. All authors read and approved the final manuscript.

## Pre-publication history

The pre-publication history for this paper can be accessed here:


